# P-1367. Changing Epidemiology of Carbapenemase-producing Carbapenem-resistant *Enterobacterales* (CRE) in Taiwan

**DOI:** 10.1093/ofid/ofae631.1544

**Published:** 2025-01-29

**Authors:** Susan Shin-Jung Lee, Hui-Ling Hsia, Yi-Ting Lee, Chii-Shiang Chen, Tsi-Shu Huang, Cheng Len Sy, Hsi-Hsun Lin

**Affiliations:** Kaohsiung Veterans General Hospital, Kaohsiung, Kaohsiung, Taiwan (Republic of China); Kaohsiung Veterans General Hospital, Kaohsiung, Kaohsiung, Taiwan (Republic of China); Kaohsiung Veterans General Hospital, Kaohsiung, Kaohsiung, Taiwan (Republic of China); Kaohsiung Veterans General Hospital, Kaohsiung, Kaohsiung, Taiwan (Republic of China); Kaohsiung Veterans General Hospital, Kaohsiung, Kaohsiung, Taiwan (Republic of China); Kaohsiung Veterans General Hospital, Kaohsiung, Kaohsiung, Taiwan (Republic of China); Kaohsiung Veterans General Hospital, Kaohsiung, Kaohsiung, Taiwan (Republic of China)

## Abstract

**Background:**

Rapid spread of carbapenemases (CP) among *Enterobacterales* require prompt identification to enable timely and appropriate treatment. This study aimed to determine the accuracy of NG-Test CARBA 5 for rapid detection of CP and to describe the epidemiology of carbapenem-resistant *Enterobacterales* (CRE) in Taiwan.

**Fig 1.**
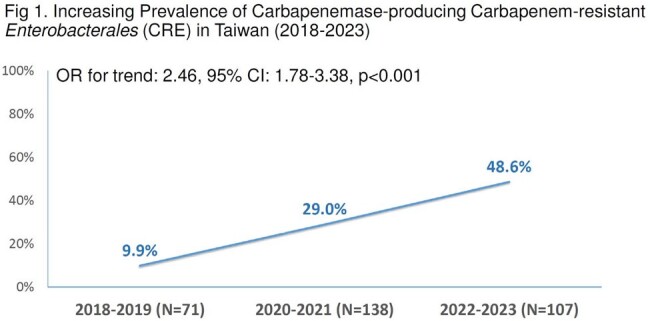
Increasing Prevalence of Carbapenemase-producing Carbapenem-resistant Enterobacterales (CRE) in Taiwan (2018-2023)

**Methods:**

316 isolates of CRE were collected from a medical center, Kaohsiung Veterans General Hospital in Taiwan during 2018 to 2023, of which 30.4% were CP producers. The NG-Test CARBA5, PCR for *bla*_KPC_, *bla*_VIM-2_, *bla*_IMP_, *bla*_NDM_, and *bla*_OXA-48-like_ genes were performed on 101 strains. Sequencing and phylogenetic analysis for *bla*_KPC_ and *bla*_NDM_ was done.

**Table 1.**
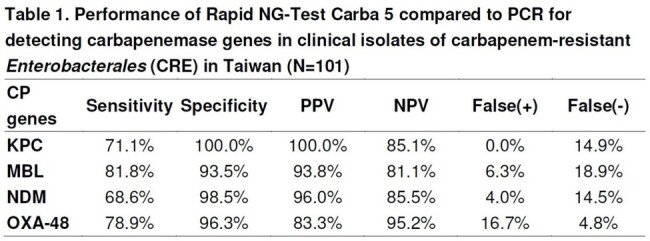
Performance of Rapid NG-Test Carba 5 compared to PCR for detecting carbapenemase genes in clinical isolates of carbapenem-resistant Enterobacterales (CRE) in Taiwan (N=101)

**Results:**

A significant increase in the prevalence of CP-producing CRE was observed during this period from 9.9% before 2020 to 29.0% in 2020-2021 and 48.6% in 2022-2023 (OR for trend: 2.46, 95% CI: 1.78-3.38, p< 0.001). CP genes was detected by PCR in 100%, however, the NG-Test CARBA 5 failed in 9 strains, with a detection rate of 91.1%. The agreement between the 2 methods were high (94.4 % for CR-*Escherichia coli*, 90.6 % for CR-*Klebsiella pneumoniae*, 87.5 % for CR-*Citrobacter freundii* and 81.8 % for CR-*Enterobacter* spp). The major CP genes in CRE was *bla*_KPC_ 38.6%, *bla*_NDM_ 34.7%, *bla*_IMP_7.6% and *bla*_OXA-48-like_ 6%. Metallo-β-lactamase (MBL) genes became prevalent over time and accounted for 54.5% of CP genes, which was mainly found in CR-*E.coli*. Highest expression of CP genes *bla*_KPC_ and *bla*_OXA-48_ was in CR-*K.pneumoniae* (52.9% and 27.5%, *bla*_NDM_ was in CR-*E.coli* (55.6%), and *bla*_IMP_ in CR-*Enterobacter* spp. (68.2%). The most common genotype of *bla*_NDM_ was *bla*_NDM-1_ (24/35, 68.6%), following by *bla*_NDM-5_ (9/35, 25.7%) and *bla*_NDM-4_ (2/35, 5.7%). All 33 *bla*_KPC_ strains were KPC-2 type.

**Fig 2.**
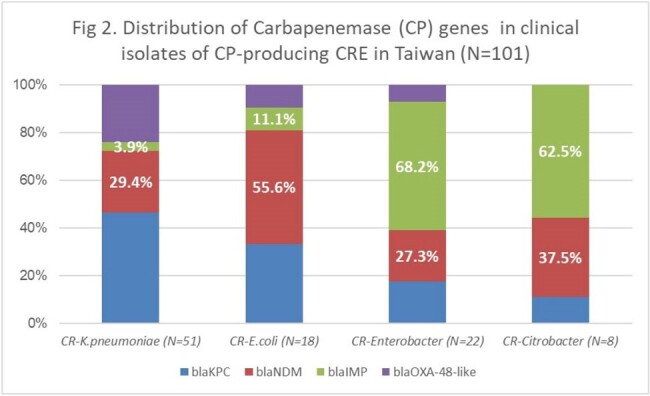
Distribution of Carbapenemase (CP) genes in clinical isolates of CP-producing CRE in Taiwan (N=101)

**Conclusion:**

Our study demonstrated that the NG-Test CARBA 5 was useful for rapid detection of CP genes, and the rising prevalence of MBL genes in Taiwan is a cause of concern due to limited treatment options.

**Disclosures:**

**All Authors**: No reported disclosures

